# Phylogenetics and genetic variation of *Heligmosomoides thomomyos* in Western pocket gophers (*Thomomys* spp.)

**DOI:** 10.21307/jofnem-2021-110

**Published:** 2022-01-13

**Authors:** Malorri R. Hughes, Alexandra A. Gibson, Emily R. Wolfe, Cecily D. Bronson, Deborah A. Duffield

**Affiliations:** 1 Department of Biology, Portland State University, 1719 SW 10th Ave, Portland, Oregon 97201; 2 One Health Institute, School of Veterinary Medicine, University of California Davis, 1 Shields Ave, Davis, CA 95616

**Keywords:** 18S rRNA, COI mtDNA, Ecology, Genetics, *Heligmosomoides thomomyos*, Systematics, Thomomys, Western pocket gophers

## Abstract

The host specificities and systematics of North American *Heligmosomoides* species remain particularly uncertain. The primary aim of this study was to verify that a species described previously based only on morphology, *H. thomomyos*, from pocket gopher (Rodentia: Geomyidae) hosts in Oregon represented a monophyletic lineage. In order to address this aspect, as well as to further understand relationships and geographic patterns, we carried out phylogenetic, genetic diversity, and population dynamic analyses using partial 18S rRNA and COI mtDNA sequences of *Heligmosomoides* specimens. Phylogenetic analyses suggested that there are likely multiple *Heligmosomoides* species present in these hosts. This was supported by the high degree of divergence and differentiation found among populations, significant population structure between locations, and a modest positive association between geographic and genetic distances. This study serves as the first molecular characterization and first phylogenetic report of *H. thomomyos*, and documents two new host records for this parasite. The relationship of *H. thomomyos* among pocket gopher hosts and to other *Heligmosomoides* species, however, warrants continued study.

The systematics and host specificities of species belonging to the genus *Heligmosomoides* (Hall, 1916) is still ambiguous (Cable et al., 2006; Behnke and Harris, 2010; Clough and Råberg, 2014) and North American forms remain especially understudied (Harris et al., 2015). Elucidating relationships within the genus are important as *Heligmosomoides* species are commonly used in immunological studies and as models for helminth infections in humans and livestock (Cable et al., 2006; Behnke and Harris, 2010; Maizels et al., 2012). Molecular studies can help quantify host specificities (Clough and Råberg, 2014) and resolve systematics-related issues by increasing the certainty of species delineations (Harris et al., 2015) as heligmosomatid species can be molecularly distinctive despite displaying morphological similarities (see Zaleśny et al., 2014). Specifically, the mitochondrial COI gene is sufficient to support *Heligmosomoides* species-level identification (Clough and Råberg, 2014).

To our knowledge, nematodes parasitizing western pocket gophers (Rodentia: Geomyidae), *Thomomys* (Wied-Neuwied, 1839) species, from Oregon have been described only using morphology (see Gardner, 1985 for a review) except for a molecular report for one species, *Trichuris fossor* (Hall, 1916) (Trichuridae) (Hughes et al., 2020). Jasmer (1980) reported the presence of an unidentified *Heligmosomoides* species (Heligmosomidae) in 23% of Botta’s pocket gophers, *Thomomys bottae* (Eydoux and Gervais, 1836), from California. Gardner and Jasmer (1983) later described this as *Heligmosomoides thomomyos* based on morphological features and suspected that *H. thomomyos* could occur in other Pacific Northwest geomyids. There has been only one other report of *H. thomomyos*, from *Thomomys bulbivorus* (Brandt, 1855; Richardson, 1829) hosts (Gardner, 1985), supporting the hypothesis that *H. thomomyos* is not host-specific to the species level.

Often, nematodes are morphologically conserved and recent molecular studies have demonstrated that many assumed monospecific species are, in fact, comprised of numerous cryptic species (Blouin, 2002). To describe with improved accuracy the biodiversity of helminths (intestinal ‘worms’) present in these hosts and to help resolve the phylogenies within Nematoda, molecular data must be accumulated. Such data can also be used to infer population dynamics and, in conjunction with DNA from the host, help understand host-parasite associations.

The primary aims of this study were to: 1) determine whether nematodes putatively identified as *H. thomomyos* from *Thomomys* hosts revealed cryptic species, 2) better define the geomyid hosts parasitized by *H. thomomyos*, and 3) serve as the first molecular report and phylogenetic study for this species. We surveyed four *Thomomys* species, *T. bottae*, *T. bulbivorus*, *T. talpoides* (Richardson, 1828), and *T. townsendii* (Bachman, 1839), that occur in Oregon for intestinal nematodes. Partial 18S rRNA and COI mtDNA sequences were used to confirm the tentative morphological identification of *Heligmosomoides* species, to evaluate the potential for cryptic species, and to elucidate intraspecific relationships. A haplotype analysis and statistical analyses were conducted to examine geographic patterns. Lastly, population differentiation statistics were calculated to better understand the genetic diversity within and among populations.

## Materials and methods

### Specimen collection

**Figure 1:**
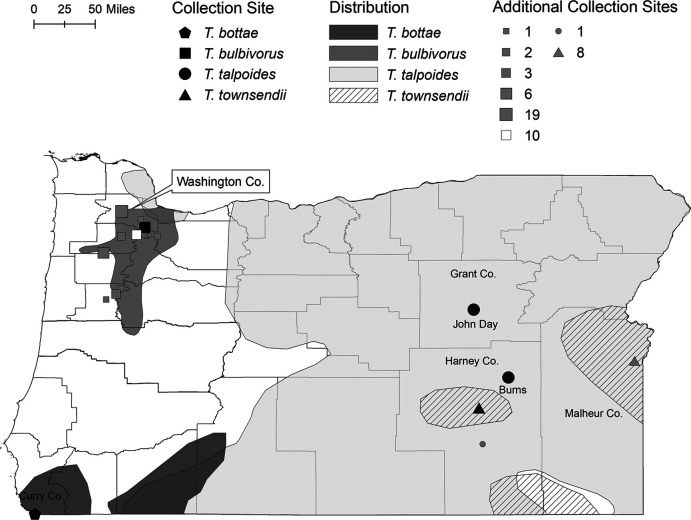
Oregon map displaying distributions and collection sites for *Thomomys* species. Black symbols represent the collection localities of sequenced *Heligmosomoides* specimens (each black symbol represents two specimens except for *T. townsendii*, which represents a single specimen). Gray symbols represent additional sites sampled where no *Heligmosomoides* were detected. From small to large, gray symbols represent sample sizes of *n* = 1, 2, 3, 6, 8, and 19. The white symbol represents a site where *Heligmosomoides* was detected, but sequencing was not performed.

One-hundred and sixteen *Thomomys* specimens were collected between March 2018 and November 2019 or salvaged from professional trappers (a subset of *T*. *bulbivorus*) (Fig. 1). Seven *T. bottae,* 83 *T. bulbivorus*, 17 *T. talpoides* (1 from Frenchglen, Harney Co., 12 from near Burns, Harney Co., and 4 from John Day, Grant Co.), and 9 *T. townsendii* (see Fig. 1) were examined for helminths following procedures outlined in Gardner and Jasmer (1983). To support field identifications of *Thomomys*, the COI gene was amplified and sequenced using the methods outlined in Spradling et al. (2004) for at least one individual per species, and the obtained sequences were compared to those available in GenBank. Helminth identification was initially based on general morphological features and previous host records (Chandler, 1945; Todd and Lepp, 1972; Jasmer, 1980; Gardner and Jasmer, 1983; Gardner, 1985). Parasites were stored in 95% EtOH and frozen prior to sequencing.

### DNA extraction, amplification, and sequencing

Two *H. thomomyos* from individual host specimens were sequenced for *T. bottae* (from Brookings, Curry Co.) and *T. bulbivorus* (from Sherwood, Washington Co.) (Fig. 1). For *T. talpoides*, four total *H. thomomyos* were sequenced from separate hosts: two from near Burns, Harney Co., and two from John Day, Grant Co. From the only infected *T. townsendii* (from Princeton, Harney Co.), one *H. thomomyos* was sequenced (*H. thomomyos* was not detected in any of the eight *T. townsendii* collected from Owyhee, Malheur Co.; Fig. 1). Before DNA isolation, specimens were transferred to fresh tubes and rinsed with distilled water to remove residual ethanol. DNA was isolated from whole worms using either the DNeasy Blood and Tissue Kit (Qiagen) (following the manufacturer’s protocols) or the Sigma-Aldrich REDExtract-N-Amp^TM^ Tissue PCR Kit. For extractions using the Sigma-Aldrich kit, we added 20  µL extraction buffer and 5  µL tissue preparation solution to each tube and ran the following protocols on a thermocycler: 10  minutes at 65°C, 10  minutes at 95°C, and 10  minutes at 10°C. We then added 30  µL neutralization solution to each tube. Amplification was carried out with PuReTaq Ready-To-Go PCR Beads (Cytiva) using the nuclear 18S ribosomal RNA primers NC18SF1 (5′-AAAGATTAAGCCATGCA-3′) and NC5BR (5′-GCAGGTTCACCTACAGAT-3′) (Chilton et al., 2006) and the mitochondrial COI primers LCO1490 (5′-GGTCAACAAATCATAAAGATATTGG-3′) and HCO2198 (5′-TAAACTTCAGGGTGACCAAAAAATCA-3′) (Folmer et al., 1994). The protocols followed for 18S rRNA PCR are outlined in Chilton et al. (2006) and the protocols followed for COI rRNA are described in Cable et al. (2006) with the exception of the annealing temperature, which was increased to 60°C. PCR success was measured on 1% agarose gels and products were purified using SPRI-magnetic beads (Elkin et al., 2001). The Center for Genome Research and Biocomputing (CGRB; Oregon State University, Corvallis, OR) processed all Sanger sequencing reactions.

### Alignment and phylogenetic analyses

Sequences were examined for quality and forward and reverse segments were combined using MEGA v. 7.0.26 (Kumar et al., 2016). Alignments were carried out in MEGA using MUSCLE. After trimming ends, 1,523 bp remained for the 18S rRNA alignment and 530 bp were used for the COI mtDNA alignment. The new sequences were deposited to GenBank under the accession numbers MZ458407-MZ458413 and MZ458119-MZ 458120 for the 18S sequences and MZ441139-MZ441147 for the COI sequences. A BLAST search against the NCBI nt database was used to identify similar sequences to include in the phylogenetic analyses. Except for the outgroup, *Tetrabothriostrongylus mackerrasae* (Mawson, 1960) (GenBank accession AJ920359), taxa were limited to representatives of Trichostrongyloidea for the 18S rRNA tree. Nine additional taxa were included in the 18S analysis (GenBank accessions AJ920355, AJ920357, AJ920358, JX877675, JX877678, LC415111, AJ920351, L04152, and AJ920350). Two North American *Heligmosomoides* species were included in the COI analyses, *H. americanus* (Durette-Desset et al., 1972) (GenBank accession KF921077) and *H. vandegrifti* (Durette-Desset and Kinsella, 2007) (GenBank accession MN928211), and *Trichostrongylus colubriformis* (Giles, 1892; Ransom, 1911) (GenBank accession MW051250) was included as the outgroup.

Mega and BEAST2 v. 2.6.0 (Bouckaert et al., 2019) were used to perform phylogenetic reconstructions. MEGA determined that the Kimura 2-parameter (K2P) with invariant sites and a gamma distribution was the best fit model for the 18S tree and that the Tamura-Nei (1993) model with invariant sites and a gamma distribution was the best fit model for the COI tree based on Bayesian information criterion. Maximum likelihood (ML) consensus trees were generated using 1,000 bootstrapping replicates. Bayesian inference (BI) analyses were prepared in BEAUti (Bouckaert et al., 2019) v.2.6.5 and completed in BEAST2 v.2.6.0. The 18S rRNA tree used the HKY model (K2P + I + G is not available in BEAST2 but the HKY model has similar parameters) and the COI tree used the TN93 model. Each analysis ran for 1 × 10^7^ generations. Tracer v. 1.7.1 (Rambaut et al., 2018) was used to assess convergence and verify each parameter had effective sample sizes (> 200 for both trees). Tree files were combined using LogCombiner v. 2.6.0 (Bouckaert et al., 2019) and maximum clade credibility (MCC) trees were made with TreeAnnotator v. 2.6.0 (Bouckaert et al., 2019) with posterior probabilities limited to 50% and a 10% burn-in percentage. FigTree v. 1.4.4 (http://tree.bio.ed.ac.uk/software/figtree/) was used to visualize the MCC tree.

### Genetic diversity analyses

The COI sequences were used to study genetic diversity and population dynamics. Pairwise distances to estimate genetic divergence were estimated in MEGA. A parsimony informative (TCS) haplotype network was constructed to visualize potential intraspecific patterns using R software (R Core Team, 2020) and the pegas package (Paradis, 2010). Overall F_ST_ values for all sequences and pairwise F_ST_ values were determined using R and the hierfstat package (Goudet, 2005). Bootstrapping (1,000 replicates) and a confidence interval of 95% was used to assess significance of pairwise F_ST_ values. An analysis of molecular variance (AMOVA; 999 permutations) was conducted using the poppr package (v. 2.9.2; Kamvar et al., 2014). A Mantel test (9,999 permutations) was performed to evaluate whether geographic distance between sites correlated with variations among the sequences.

## Results

*Heligmosomoides* were found in 23 (21.70%) of the examined *Thomomys*. Five (71.43%) *T. bottae*, five (6.02%) *T. bulbivorus*, 12 (9 from near Burns and 3 from John Day) (64.7%) *T. talpoides*, and one (11.11%) *T. townsendii* were infected (Fig. 1). A subset of the detected *Heligmosomoides* were sequenced (Fig. 1). Intensity (number of individuals per host) of infections ranged from 1 to 41 (xˉ = 6.9). *Heligmosomoides* infections were not detected in the majority (90.9%) of *T. bulbivorus* locations sampled (Fig. 1). Field identifications of *T. bulbivorus* were supported genetically (> 98% COI gene sequence identity); *T. bottae* and *T. talpoides* field identifications were weakly supported (85.6–89.8% COI gene sequence identity); and confirmation of *T. townsendii* were not possible due to a lack of overlapping sequence availability in GenBank. Despite the lack of genetic support for some pocket gopher species identifications, we used published *Thomomys* distributions and morphological characteristics (Verts and Carraway, 1998) to assign the field identifications and maintain these identifiers throughout. *Thomomys* COI sequences were deposited in GenBank under the accession numbers OK501245–OK501263.

### 18S phylogeny results

**Figure 2:**
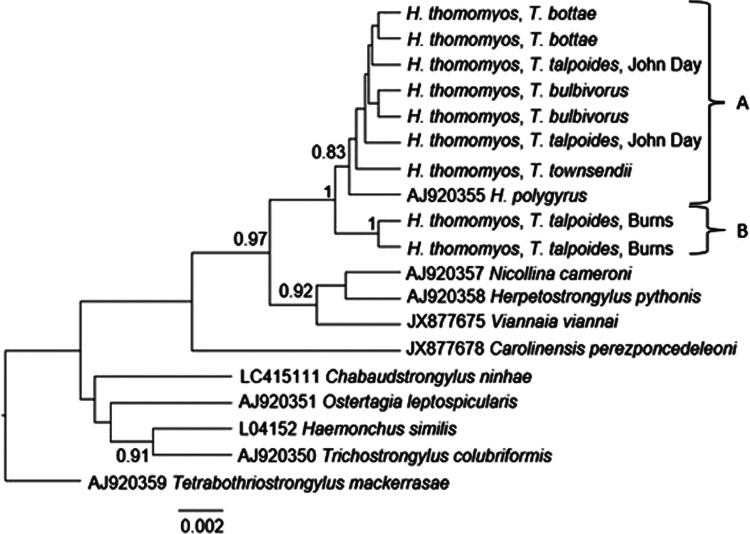
Bayesian inference tree constructed from 18S rRNA sequences based on the HKY model. Posterior probabilities > 70% are shown near nodes. For new sequences, the host species is listed, and, for *T. talpoides*, the nearest township is specified. Scale is in substitutions per site.

All *Heligmosomoides* species (from this study and the GenBank reference sequence) formed a monophyletic group with high posterior probability support (100%) in the BI tree (Fig. 2). However, due to the position of *H. polygyrus* (Dujardin, 1845) (Clade A), *H. thomomyos* was paraphyletic (Fig. 2). Within *H. thomomyos*, two distinct clades were supported with high posterior probabilities (100% and 83%). The majority of our sequences were most similar to the *H. polygyrus* sequence from GenBank (accession AJ920355), as evidenced by its placement within Clade A (Fig. 2). However, posterior probabilities within Clade A were too low to infer finer-scale relationships using the 18S gene. Two *Heligmosomoides* sequences from the *T. talpoides* hosts collected near Burns formed the second clade (Clade B; Fig. 2). These results were also achieved using the ML method (not shown), which reflected similar relationships and nodal support values.

### COI phylogeny results

**Figure 3:**
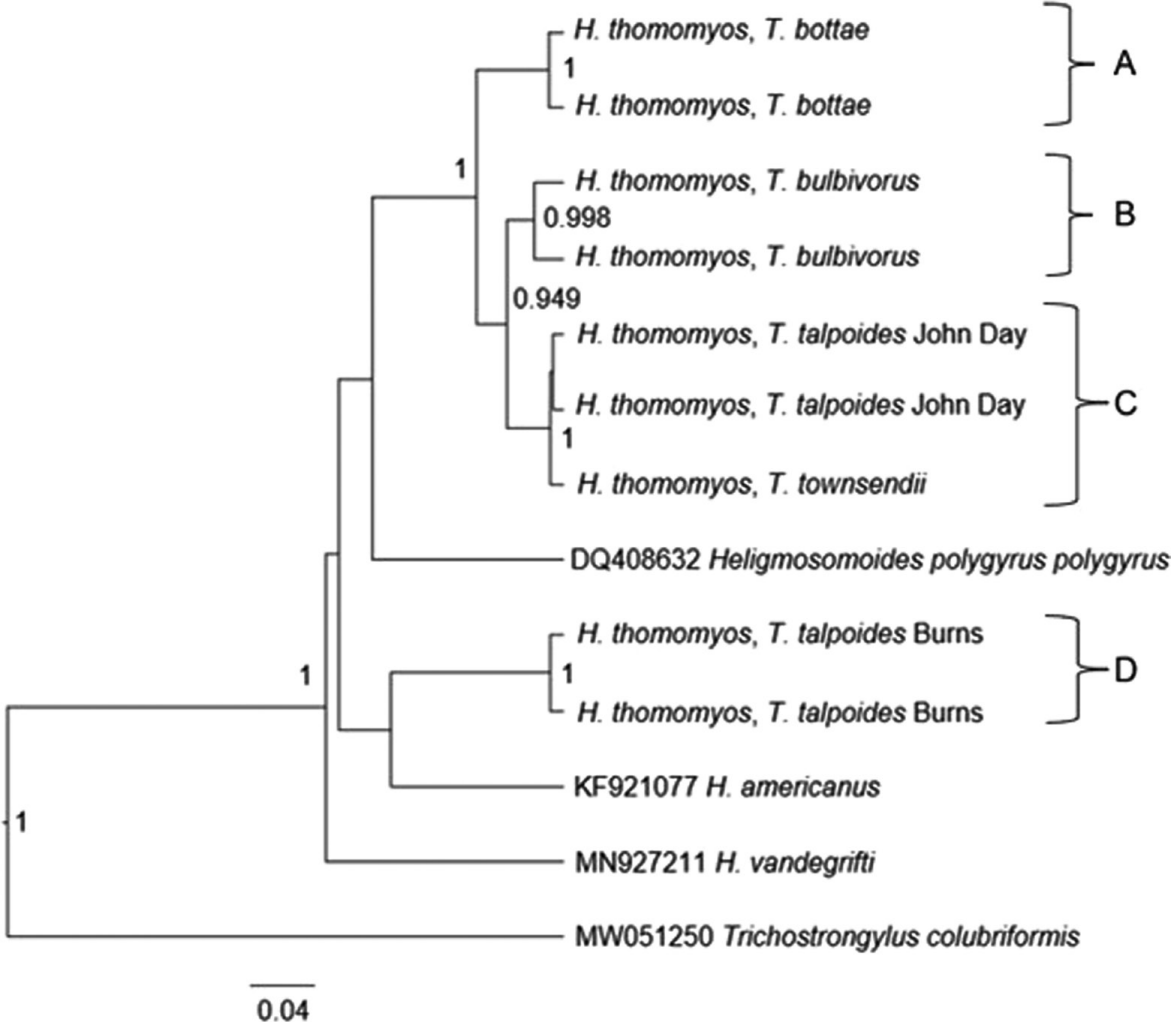
Bayesian inference method tree constructed with COI mtDNA sequences and based on the TN93 + I + G model. Posterior probabilities < 70% are not shown, those > 70% are shown near nodes. Sequences from this study list the host name and, for *T. talpoides* specimens, the nearest township in Oregon. Scale is in substitutions per site.

Similar to the 18S tree, all samples belonging to the *Heligmosomoides* genus formed a monophyletic group in the COI BI tree (Fig. 3). However, our *H. thomomyos* samples were paraphyletic, owing to the closer relationship of the *T. talpoides* Burns samples to the *H. americanus* sequence (Fig. 3). The COI tree did yield a more detailed perspective on intrageneric relationships. Four distinct clades were supported with high posterior probabilities (99–100%) and, in every instance, *Heligmosomoides* sequences from the same location were monophyletic. Clades A and B each contained sequences from only a single host species, *T. bottae* and *T. bulbivorus*, respectively (Fig. 3). The *Heligmosomoides* sequence from *T. townsendii* was sister to those from the John Day *T. talpoides* hosts, and together these three sequences formed Clade C with a 100% posterior probability support (Fig. 3). Clade D was comprised of *Heligmosomoides* sequences from *T. talpoides* from Burns, yet these clustered with *H. americanus* (GenBank accession KF921077) rather than other *Heligmosomoides* from this study. The topology of the BI tree was identical to that of a ML BS consensus tree (1,000 replicates; not shown) except for the placement of the outgroups in relation to Clade D.

### Genetic diversity results

COI pairwise divergence results are shown in Table 1. The average COI divergence across all *Heligmosomoides* samples was 6.2%. Within *H. thomomyos* sequences obtained from the same host species at the same location, the average genetic divergence was 1.5%, the greatest genetic distance (2.7%) was observed between sequences from *T. bulbivorus* hosts (Clade B) from Washington Co., and the lowest genetic divergence (0.8%) was observed between sequences from *T. talpoides* hosts from John Day (Clade A). Across different collection sites, the average genetic divergence was 6.4%, the greatest genetic distance (11.3%) was observed between a sequence from a *T. talpoides* host from Burns (Clade D) and a sequence from a *T. bottae* host (Clade A), and the lowest genetic divergence (1.1%) was observed between a sequence from a *T. talpoides* host from John Day (Clade A) and a sequence from a *T. townsendii* host (Clade B). Consistent with COI clade topology from the BI tree, *H. thomomyos* sequences from Clade D were the most divergent, on average, from the other *H. thomomyos* clades.

**Table 1.** Above the diagonal are the average percentages and, in parenthesis, ranges of evolutionary pairwise distances among *H. thomomyos* COI mtDNA sequences.

	1	2	3	4	5
1. *H. thomomyos*, *T. bottae*, Clade A	1.5% –	xˉ = 7.0% (6.5–7.6%)	xˉ = 10.8% (10.4–11.3%)	xˉ = 6.2% (6.0–6.4%)	xˉ = 6.1% (6.0–6.2%)
2. *H. thomomyos*, *T. bulbivorus*, Clade B	0.259* (0.174, 0.343)	2.7% –	xˉ = 6.8% (6.2–7.3%)	xˉ = 4.3% (3.9–4.7%)	xˉ = 4.3% (3.9–4.7%)
3. *H. thomomyos*, *T. talpoides*, Burns, Clade D	0.350* (0.291, 0.406)	0.345* (0.279, 0.407)	1.1% –	xˉ = 9.1% (8.7–9.4%)	xˉ = 9.1% (8.9–9.2%)
4. *H. thomomyos*, *T. talpoides*, John Day, Clade C	0.413* (0.333, 0.474)	0.284* (0.150, 0.393)	0.458* (0.414, 0.492)	0.8% –	xˉ = 1.1% (1.1–1.1%)
5. *H. thomomyos*, *T. townsendii*, Clade C	0.384* (0.248, 0.405)	0.121 (‒0.076, 0.258)	0.444* (0.339, 0.425)	0.063 (‒0.429, 0.600)	–

Notes: Below the diagonal are pairwise F_ST_ comparisons. Upper and lower confidence intervals are shown in parenthesis. F_ST_ values significantly different from 0 (determined using 1,000 bootstrap replicates) are indicated by an asterisk. Clade information is provided in alignment with Fig. 3.

**Figure 4:**
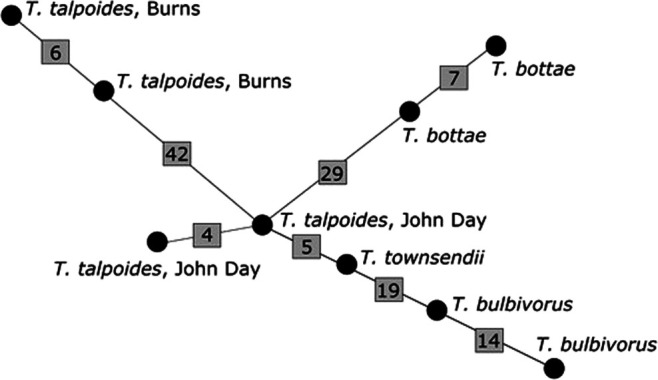
TCS haplotype network constructed from nine COI mtDNA sequences from this study. Each sequence represented a unique haplotype, which are represented as circles. The number of mutations are shown along branches in gray squares.

Each of the nine COI sequences represented a unique haplotype in our network results, including those from the same localities (Fig. 4). The average number of mutational steps was 15.75. The highest observed number of mutational steps (42) was between specimens from a *T. talpoides* host from John Day and a *T. talpoides* host from Burns while the least mutational steps (4) was between the two *T. talpoides* from John Day. These results aligned with our observed pairwise distance values (Table 1) and clade groupings in our COI tree (Fig. 3).

The overall F_ST_ value was 0.3031 for all *H. thomomyos* COI samples. Pairwise F_ST_ values are shown in Table 1. While the AMOVA detected significant population structure between locations (i.e., collection sites; p-value = 0.003; Φ = 0.815; 35.11% of the variation), most of the variation was within samples across all locations (113.87%). However, it is worth noting here that the >100% variation is the result of a negative value for within sample population structure, which is almost certainly driven by low or uneven sample sizes among populations due to sampling limitations. Consequently, it should be interpreted as a statistical artifact indicative of unevenly distributed genetic variation among the sampled populations (Meirmans, 2007). Finally, we also found a modest positive association between geographic and genetic distances for the COI gene (Mantel test, *r* = 0.472, *p* = 0.0198).

## Discussion

This serves as the first study to document the presence of *Heligmosomoides* species in geomyids using molecular markers. This study provides further support for the cryptic diversity of nematodes and verifies that morphologically identified *H. thomomyos* from this study actually represent multiple distinctive lineages. In addition, we documented new host records for *H. thomomyos* in two pocket gopher species, *T. townsendii* and *T. talpoides*, with the latter possibly host to multiple *Heligmosomoides* species.

We predicted that *H. thomomyos* sequences would form a monophyletic clade in both the 18S and COI analyses, and that sequences from the same host species would be sister taxa in the COI analysis. The 18S BI tree deviated from this expectation, as the placement of the *H. polygyrus* sequence from GenBank created a paraphyletic relationship among our samples. Furthermore, low support values (posterior probabilities < 50%) within Clade A of the 18S tree did not allow for finer-scale resolution among our *H. thomomyos* sequences. Given the slow mutation rate of the 18S gene in comparison to the COI gene, this analysis was not expected to yield interesting results, given we had morphologically identified all nematodes as a single species, *H. thomomyos*. Thus, the position of *H. thomomyos* from Burns was surprising. The COI BI tree also revealed a paraphyletic relationship among our *H. thomomyos* samples. However, the sister taxa relationships of *H. thomomyos* from the same host species and from the same collection localities in the COI tree aligned more with the anticipated relationships. Based on our analyses, *H. thomomyos* could be paraphyletic or, more likely, multiple cryptic *Heligmosomoides* species could be present in these *Thomomys* hosts. Other genes, especially the ITS1 and ITS2 regions, the 5.8S rRNA gene, and the 28S rRNA gene, as well as a thorough morphological analysis, should be evaluated to definitively determine the true *Heligmosomoides* diversity present. Further studies that survey a broader distribution would also help establish host specificities and systematics of the *Heligmosomoides* complex within rodent hosts (Clough and Råberg, 2014). Given that *Thomomys* taxonomy is not fully resolved (especially in the *Megascapheus* subgenus; see Trujano-Alvarez and Álvarez-Castañeda, 2013; Mathis et al., 2014), the high number of recognized subspecies within a majority of *Thomomys* spp. (Hall, 1981; Trujano-Alvarez and Álvarez-Castañeda, 2013), and the extremely high mitochondrial genetic variation documented within *Thomomys* (Mathis et al., 2013; Mathis et al., 2014), further studies that better elucidate *Thomomys* diversity, especially regarding *T. talpoides*, could be equally helpful when inferring host specificity and systematics of *Heligmosomoides.*

Based on the pairwise evolutionary distances, there was a high level of divergence within the COI gene as expected based on the known mutation rate in this gene (Blouin et al., 1998; Denver et al., 2000) in comparison to 18S. Pairwise comparisons within *H. thomomyos* from the same collection site ranged from 0.8 to 2.7%, which is comparable to pairwise comparisons observed within *H. polygyrus* isolates from the UK (1–6%; Cable et al., 2006) and within *H. polygyrus* clades identified across the Palearctic (2.36%; Nieberding et al., 2005). However, the degree of divergence observed in our among-site *H. thomomyos* comparisons are in agreement with some of the pairwise distances observed between various *Heligmosomoides* species examined within Cable et al. (2006) (9.5–55.5%). Blouin (2002) proposed that mitochondrial sequence differences greater than 10% were likely sufficient to delineate between species of nematodes. Several of the differences we report are close to or exceed this threshold (Table 1).

The COI haplotype network further supports evidence of divergence within our *H. thomomyos* sequences, demonstrating that a high number of mutational steps separate many of the *H. thomomyos* sequenced from different hosts and collection sites. Not surprisingly, each sequence did represent a unique haplotype; however, it is the amount of divergence across clades that was most intriguing. The 42 mutational steps separating sequences from *T. talpoides* collected near Burns from the other *H. thomomyos* exceeds the 39 substitutions observed by Cable et al. (2006) in their comparisons of different *Heligmosomoides* species across the UK, USA, and Guernsey. Additionally, Cable et al. (2006) also observed 39 substitutions between *H. polygyrus* isolates from the UK and *Heligmosomum mixtum* (Schulz, 1954) from Poland, while intraspecies comparisons of *H. polygyrus* revealed that only 1 to 8 substitutions separated individuals within this taxon. Furthermore, a Palearctic-wide phylogeographic analysis of *H. polygyrus* cytochrome b sequences observed similar divergence (18–35 mutational steps) across the five identified haplotype groups, whereas intra-clade divergence averaged 6.3 mutational steps (Nieberding et al., 2005). Given that our analysis was confined to the state of Oregon, as opposed to across continents, and that 4 to 14 (average = 7.75) substitutions separated our *H. thomomyos* sampled from the same location, whereas 5 to 163 (average = 46.58) substitutions separated our *H. thomomyos* from different locations across the state, our results provide further support that these nematodes are highly cryptic in nature, and indicates that more than one *Heligmosomoides* species is likely present in our analysis.

The overall F_ST_ value and most of the pairwise F_ST_ values were high for the COI analysis (xˉ = 0.312; 0.063–0.458), indicating a high degree of divergence and genetic differentiation among populations. Future studies incorporating larger sample sizes would help lend further support to these observed F_ST_ values; however, the conclusions drawn from this analysis do align with the results of our phylogenetic and haplotype network analyses. Rates of gene flow for vertebrate nematode parasites is most influenced by life history traits and host mobility (Nieberding et al., 2005; Wu et al., 2009). Pocket gophers remain in isolated pockets throughout their distributions (Light and Hafner, 2007) and their populations can exhibit low levels of gene flow (Smith, 1998). Given this, it is not surprising that there was a low amount of gene flow and high degree of differentiation detected in the COI sequences among these *H. thomomyos* populations. These findings are also consistent with the COI tree clades, which revealed sister taxa relationships of *H. thomomyos* from hosts from the same collection sites. Host subspecies or inter-host relationships (see Belfiore et al., 2008 and Smith, 1998) could be shaping the differentiation we observed, given that *Thomomys* taxonomy is not fully resolved for all species (Trujano-Alvarez and Álvarez-Castañeda, 2013; Mathis et al., 2014). Based on species distribution records, the *T. talpoides* specimens from Burns and John Day do represent different subspecies (Verts and Carraway, 1998) which could be contributing to the high divergence observed. However, it is possible that both of the *Heligmosomoides* species we believe to have documented within *T. talpoides* were present at both locations, but due to our small sample sizes we did not detect them at each site. Likewise, the direct lifecycle of *Heligmosomoides* species and ecological variation could contribute to the observed genetic differentiation.

Most population-level helminth studies reveal a high degree of diversity within localities and “extremely low differentiation among localities”, signifying a high amount of gene flow (Nieberding et al., 2005). The majority of these studies were on “parasites of humans, domestic animals, commensals or game species”, thus this trend may not hold true for helminths infecting wildlife populations (Nieberding et al., 2005). Typically, higher within sample variation than between population variation is indicative of high gene flow and lack of population substructure. This was not the case for the rapidly evolving COI gene, for which we found significant population differentiation among all collection sites (*F*_ST_ > 0.05) as well as a significant correlation between increasing geographic and genetic distances. The high within sample variation detected in the COI AMOVA could be caused by small sample sizes (see Wasike et al., 2005), thus replicating this analysis with larger sample sizes could be worthwhile. Additionally, given that there are likely multiple, cryptic *Heligmosomoides* species contained within this analysis, it is possible that the population differentiation observed is actually correlated with speciation within *Heligmosomoides*, rather than within the *H. thomomyos* taxon. Thus, further research to accurately delineate *Heligmosomoides* species (morphologically and genetically) is necessary to support the population differentiation observed here.

Given that helminths are extremely common in rodent hosts (Spickett et al., 2017), it is not surprising that we found evidence that more than one species may be present in these hosts based on an analysis of nuclear and mitochondrial molecular markers. The nematodes identified in this study parasitize geomyid hosts, but cryptic *Heligmosomoides* from other rodent hosts (e.g., field mice) have been documented previously (Cable et al., 2006; Zaleśny et al., 2014). Our phylogenetic analyses suggest that further studies on *Heligmosomoides* in geomyid hosts will help resolve systematics and population structure with potential carry-over implications for similar host–parasite interactions, such as those infecting humans and livestock or those used in immunological studies. Broader sampling efforts could answer questions regarding variation in infections among hosts and geographic regions—the moderate positive correlation between genetic and geographic distances we found implies that experimental designs should cover significant portions of host species’ ranges to fully capture patterns in population genetics. Finally, we show the utility in using multiple molecular markers (i.e., for an orthologous nuclear gene and for a less conserved mitochondrial gene) to resolve phylogenetics and population structure.
